# Abnormal frontostriatal activity in recently abstinent cocaine users during implicit moral processing

**DOI:** 10.3389/fnhum.2015.00565

**Published:** 2015-10-16

**Authors:** Brendan M. Caldwell, Carla L. Harenski, Keith A. Harenski, Samantha J. Fede, Vaughn R. Steele, Michael R. Koenigs, Kent A. Kiehl

**Affiliations:** ^1^The Mind Research Network and Lovelace Biomedical and Environmental Research InstituteAlbuquerque, NM, USA; ^2^Department of Psychology, University of New MexicoAlbuquerque, NM, USA; ^3^Intramural Research Program, Neuroimaging Research Branch, National Institute of Drug Abuse, National Institutes of HealthBaltimore, MD, USA; ^4^Department of Psychiatry, University of Wisconsin-MadisonMadison, WI, USA

**Keywords:** Cocaine, Moral Intuition, Social Cognition, fMRI, Psychopathy

## Abstract

Investigations into the neurobiology of moral cognition are often done by examining clinical populations characterized by diminished moral emotions and a proclivity toward immoral behavior. Psychopathy is the most common disorder studied for this purpose. Although cocaine abuse is highly co-morbid with psychopathy and cocaine-dependent individuals exhibit many of the same abnormalities in socio-affective processing as psychopaths, this population has received relatively little attention in moral psychology. To address this issue, the authors used functional magnetic resonance imaging (fMRI) to record hemodynamic activity in 306 incarcerated male adults, stratified into regular cocaine users (*n* = 87) and a matched sample of non-cocaine users (*n* = 87), while viewing pictures that did or did not depict immoral actions and determining whether each depicted scenario occurred indoors or outdoors. Consistent with expectations, cocaine users showed abnormal neural activity in several frontostriatial regions during implicit moral picture processing compared to their non-cocaine using peers. This included reduced moral/non-moral picture discrimination in the vACC, vmPFC, lOFC, and left vSTR. Additionally, psychopathy was negatively correlated with activity in an overlapping region of the ACC and right lateralized vSTR. These results suggest that regular cocaine abuse may be associated with affective deficits which can impact relatively high-level processes like moral cognition.

## Introduction

“Moral cognition” refers to a set of complex processes giving rise to, and including, an individuals' judgments about the moral status of a person or action in a particular scenario. Academic interest in this aspect of our social world has been longstanding, but recent decades have seen a rapid expansion of moral psychological research into a myriad of scientific disciplines. Cognitive neuroscience is one of the more recent members— employing newly emerging neuroimaging techniques to provide novel insights into the neural circuitry engaged by moral cognition.

Many differing theoretical perspectives on moral cognition have followed, but one aspect which is widely shared amongst them is the bifurcation of moral decision-making into two relatively distinct, though highly interdependent, processes. The first, which we will refer to as “moral intuition,” consists of relatively automatic and reflexive operations through which an individual attends and responds to the morally-salient aspects of a given stimulus. “Moral deliberation,” by contrast, denotes more deliberative and cognitively taxing processes (e.g., evaluating the moral appropriateness of stimuli and identifying morally appropriate actions; Harenski et al., 2010a). The precise nature and role of these two processes is controversial (Haidt, 2001; Pizarro and Bloom, 2003; Greene et al., 2004; Hauser, 2006), but it is widely assumed that both play a significant role in moral cognition. Studying these two processes in isolation has subsequently become a major focus in moral cognition research (for review see Hauser, 2006).

The primary way neuroimaging studies have begun to tease apart these processes is by subtle task manipulations. To date, the majority of neuroimaging studies have employed “explicit” moral judgment tasks, in which participants are shown morally-salient stimuli and asked to rate their moral appropriateness. Because performance of these tasks requires both attendance to and reflective consideration of the moral information presented, the resultant neural activity likely represents both moral intuition and deliberation. The brain regions most consistently activated during explicit moral judgment tasks include the ventromedial prefrontal cortex (vmPFC), dorsolateral prefrontal cortex (dlPFC), temporo-parietal junction (TPJ), cingulate cortices (ACC and PCC), and amygdala (Amyg; for review see Fumagalli and Priori, 2012).

In contrast, a minority of neuroimaging studies have utilized ‘implicit’ moral tasks, in which morally-salient stimuli are presented but explicit moral judgments are not required. Tasks of this sort are thought to maintain the reflexive, perceptual aspects of moral cognition while minimizing its deliberative components. These studies have generally identified the TPJ and PCC as being central to the earliest stages of moral cognition (Harenski and Hamann, 2006; Moll et al., 2007; Schiach-Borg et al., 2008; Harenski et al., 2010a; Decety and Cacioppo, 2012).

Over the past decade, researchers have begun examining the neurobiology of moral cognition in clinical populations characterized by diminished moral emotions (e.g., guilt, empathy) and a proclivity toward immoral behavior. Psychopathy is the most common disorder studied for this purpose—a personality disorder typified by a suite of interpersonal, affective, and behavioral characteristics including grandiosity, callousness, and lack of empathy (Cleckley, 1976; Hare, 2003; Hare and Neumann, 2008). Individuals at the high end of this spectrum are targeted because they demonstrate a marked moral insensitivity and are disproportionately responsible for repetitive crime and violence in society (Hart and Hare, 1989; Harris et al., 1991; Hemphill et al., 1998).

Substance abuse is, in many ways, comparable to psychopathy, but has received much less attention in moral psychology. Substance abuse and psychopathy are highly co-morbid (Smith and Newman, 1990; Walsh et al., 2007) and substance abusers have consistently shown social and affective deficits compared to healthy controls (Aguilar de Arcos et al., 2005; Verdejo-García et al., 2007; Fernández-Serrano et al., 2010; Volkow et al., 2011).

After cannabis, cocaine is the second most widely abused illicit drug in the United States, with an estimated 4.4 million users in the United States as of 2013 (NSDUH). Moreover, both current and former cocaine-dependent individuals exhibit many of the same abnormalities in socio-affective processing as psychopaths. Psychopaths exhibit significant deficits in affective empathy and facial affect recognition (for review see Brook et al., 2013). Cocaine abusers, both active and abstinent, show similar deficits in emotion recognition, with fear being the most consistently misidentified emotional expression (Foisy et al., 2005; Fox et al., 2007, 2011; Kemmis et al., 2007; Verdejo-García et al., 2007; Fernández-Serrano et al., 2010; Morgan and Marshall, 2013). Deficits in social cognition have been observed as well, with cocaine abusers exhibiting impaired empathy (Ferrari et al., 2014; Hulka et al., 2014; Preller et al., 2014).

Social, affective, and moral cognition are thought to be highly interrelated at the neural level (Young and Dungan, 2012). As such, the similarities in social and affective abnormalities between psychopaths and cocaine abusers may very well extend into the moral domain. Although researchers have only recently begun to examine moral cognition amongst current and former substance abusers, early results have supported this conclusion. Similar to psychopaths, polysubstance dependent individuals have been found to make more “utilitarian” moral judgments than healthy controls (Carmona-Perera et al., 2012; Kornreich et al., 2013). The only neuroimaging study comparing moral cognition in cocaine-dependent and non-dependent individuals found that cocaine-dependent individuals did show reduced activity in paralimbic structures such as the dorsal ACC, PAG, and anterior insula while making moral judgments (Verdejo-García et al., 2012).

The somatic marker theory of addiction (Verdejo-García and Bechara, 2009) is a promising framework for conceptualizing the relationship between substance abuse and social cognition. This model theorizes two distinct, though highly interdependent, neural systems which play a role in any sort of decision-making. The core structure of the “impulsive neural system” is the amygdala, which couples stimuli with their associated somatic state. That somatic state is generated by effector structures such as the hypothalamus, autonomic brainstem nuclei, ventral striatum, and periaqueductal gray. The core structure of the “reflective neural system” is the vmPFC, which integrates emotional information—generated by the insula and PCC—and cognitive information—generated by the dlPFC and hippocampus. Drug addiction is thought to be the result of a hyperactive impulsive system, which weakens the regulatory power of the reflective system.

Etiologically, this is likely the result of the abused drug “hijacking” the normal reward circuitry, leading to an over-valuation of the drug at a neurological level. Current and abstinent stimulant addicts have demonstrated exaggerated autonomic responses to cues related to the substances they abuse(d) and a muted response to all non-drug affective stimuli (e.g., cues related to food, sex, etc…; Aguilar de Arcos et al., 2005; Asensio et al., 2010; Kim et al., 2011b). Blunted positive responses to pro-social interactions and blunted negative responses to socially aversive experiences could account for the deficits in social cognition and deleterious social behavior of stimulant-dependent individuals (Volkow et al., 2011). Indeed, a few studies have found that the emotional and decision-making deficits seen in cocaine-dependent individuals are linked to the real-world social dysfunction of addicts (Cunha et al., 2011; Preller et al., 2014).

The extant neuroimaging studies on social and moral cognition in cocaine abusers have two notable limitations. First, none of these studies have examined moral intuition amongst cocaine abusers. Many real-world moral evaluations occur spontaneously upon the presentation of a morally salient social situation. An examination of moral intuition specifically may therefore provide us with a more ecologically rounded assessment of moral cognition. Secondly, none of these substance abuse studies examined the potential impact of comorbid psychopathy. The overlapping prevalence and associated neuropsychological deficits exhibited by these two disorders suggests that they may confound one another.

Here we used functional magnetic resonance imaging (fMRI) to evaluate brain activity during the performance of a moral intuitive processing task by individuals with varying levels of psychopathy and (past) cocaine use. Using the Mind Research Network's mobile MRI system, incarcerated participants were scanned while viewing pictures that did or did not contain moral violations (e.g., a hand breaking into a house vs. a mutilated hand), as well as affectively neutral pictures (e.g., a hand being fingerprinted), and judging whether each picture depicted a scene occurring indoors or outdoors. Participants were therefore exposed to morally salient stimuli, but were not made aware of the moral/non-moral picture distinction (Harenski et al., 2010a).

Integrating the findings of prior studies, we predicted that former cocaine users would exhibit reduced hemodynamic activity compared to non-cocaine users during the implicit processing of moral stimuli. Given the theorized etiology for such an effect, we predicted that abnormalities amongst cocaine users would be located in brain regions along the dopaminergic pathway normally used for reward processing—including the striatum, ACC and mPFC. Additionally, we hypothesized that cocaine users high in psychopathic traits would exhibit greater abnormalities in these areas compared to cocaine users lower in psychopathic traits.

## Materials and methods

### Participants

This study included 316 incarcerated male volunteers recruited from correctional facilities in New Mexico and Wisconsin. Inclusion criteria were: age between 18 and 50, reading level above 4th grade, IQ above 75, no history of seizures, no current Diagnostic and Statistical Manual of Mental Disorders (4th Edn.; American Psychiatric Association, 1994) Axis I diagnosis (excluding substance abuse), and no lifetime history of a psychotic disorder in self or first-degree relative. Incarcerated participants were in a controlled environment with restricted access to illicit drugs, and all reported to be abstinent from substance use for at least 1 month prior to testing. Ten incarcerated participants were excluded from analysis due to excessive motion during scanning. The final sample included 311 incarcerated participants. Incarcerated participants were included in the “Cocaine User” (CU) group if they had regularly used cocaine (three or more times per week) for any duration of time (*n* = 87). The remaining incarcerated participants were assigned to the Non-Cocaine Using (NCU) group (*n* = 219). Additionally, a subset of the NCU group was matched with the CU group on age, IQ, psychopathy and severity of use for substances other than cocaine. This subset will be referred to as the Matched Non-Cocaine Users (mNCU). Demographic information on each group is provided in Table 1. Group comparisons on demographic variables between these three subsamples are provided in Table 2.

**Table 1:** **Demographic information of incarcerated cocaine users and non-cocaine users**.

**Variable**	***NCU (n = 219)***		***CU(n = 87)***		***mNCU (n = 87)***	
	**Mean**	***SD***	**Mean**	***SD***	**Mean**	***SD***
Age	32.15	8.07	34.9	8.08	33.59	8.90
IQ	98.66	13.66	100.62	13.09	99.34	13.27
**PSYCHOPATHY**						
PCLR Total	22.265	7.4594	24.34	6.11	23.31	7.82
PCLR Factor 1	8.037	3.7866	8.05	3.73	8.05	3.99
PCLR Factor 2	12.186	4.2716	13.86	3.13	13.04	4.29
**SUBSTANCE USE HISTORY**						
Alcohol	0.105	0.148	0.205	0.195	0.17	0.18
Cannabis	0.199	0.198	0.351	0.208	0.31	0.21
Heroin	0.061	0.054	0.122	0.105	0.02	0.09
Other Opiates	0.018	0.067	0.056	0.101	0.03	0.09
Cocaine	0.000	0.000	0.136	0.141	0.00	0.00
Methamphetamine	0.007	0.043	0.051	0.128	0.01	0.07
Other Amphetamines	0.007	0.034	0.026	0.095	0.01	0.05
Nicotine	0.248	0.223	0.408	0.194	0.36	0.23
	***N***	**%**	***N***	**%**	***N***	**%**
**RACE**						
American Indian/Alaskan Native	12	5.5	0	0	7	8
Asian	1	0.5	0	0	1	1.1
Black	73	33.3	13	14.9	27	31
Native Hawaiian/Pacific Islander	3	1.4	0	0	0	0
White	111	50.7	59	67.8	44	50.6
Other/Decline	19	8.7	15	17.2	8	9.2
**ETHNICITY**						
Not Hispanic	193	88.1	64	73.6	77	88.5
Hispanic	26	11.9	23	26.4	10	11.5
**HANDEDNESS**						
Right	184	84	78	89.7	73	83.9
Left	26	11.9	7	8	11	12.6
Ambidextrous	9	41	2	2.3	3	3.4

*For each substance, substance use history is represented by the proportion of an individual's lifetime the substance was being used regularly (e.g., Alcohol Use = 0.1044 means the participant used alcohol regularly for 10.44% of his life)*.

**Table 2:** **Group comparisons of demographic information for incarcerated cocaine users and non-cocaine users**.

**Variable**	***CU*** **vs**. ***NCU***			***CU*** **vs**. ***mNCU***		
	***t***	***df***	***p***	***t***	***df***	***p***
Age	2.69	304	0.008	1.02	172	0.312
IQ	1.15	304	0.252	0.64	172	0.523
**PSYCHOPATHY**						
PCLR Total	2.51	191.4	0.013	0.97	163	0.336
PCLR Factor 1	0.02	304	0.984	0.00	172	1.000
PCLR Factor 2	3.74	217.33	0.000	1.42	152	0.158
**SUBSTANCE USE HISTORY**						
Alcohol	4.34	127.44	0.000	1.16	172	0.246
Cannabis	5.98	304	0.000	1.60	172	0.112
Heroin	3.02	107.90	0.003	1.45	169	0.150
Other Opiates	3.24	116.88	0.002	1.43	172	0.155
Cocaine	9.00	86	0.000	9.12	172	0.000
Methamphetamine	3.14	93.791	0.002	2.37	172	0.019
Other Amphetamines	1.82	94.704	0.073	1.08	172	0.283
Nicotine	6.22	180.47	0.000	1.62	172	0.107
	**χ**^2^	***df***	***p***	**χ**^2^	***df***	***p***
Race	21.25001	5	0.001	17.2149	4	0.002
Ethnicity	9.82098	1	0.002	6.319794	1	0.012
Handedness	1.644219	2	0.440	1.25	2	0.534

*For each substance, substance use history is represented by the proportion of an individual's lifetime the substance was being used regularly*.

In order to generate task-specific ROIs, fMRI data was analyzed from a separate sample of 36 non-incarcerated male volunteers. Non-incarcerated participants were held to the same inclusion criteria, with the additional requirement that non-incarcerated participants be excluded if they had any history of an alcohol or drug use disorder.

### Assessments

Psychopathy was assessed in all incarcerated participants using the Hare Psychopathy Checklist-Revised (PCL-R; Hare, 2003), which is a reliable and valid instrument for assessing psychopathy in incarcerated populations (Hare, 1980, 1996; Hart and Hare, 1989; Fulero, 1996). Trained researchers conducted semi-structured interviews covering topics such as school, employment, relationships, family, and criminal activity. Interview information was also corroborated by a review of the participant's institutional records. The PCL-R contains 20 items designed to measure the personality and behavioral characteristics of psychopathy. Each of these items is scored on a 3-point scale and total scores range from 0 to 40. Factor analyses of the PCL-R have suggested that its items load onto two correlated factors. Factor 1 contains items having to do with the affective/interpersonal features of psychopathy, while Factor 2 consists of those features of psychopathy associated with an impulsive, antisocial, and unstable lifestyle (Hare, 2003).

All participants completed the Structured Clinical Interview for DSM-IV Disorders (SCID; First et al., 2002) with a trained research assistant, to assess past and present Axis I and II disorders. This included the drug use disorder screening questionnaire and module, which were used to evaluate alcohol and drug disorder histories for exclusion purposes (non-incarcerated participants) and identify prior substance use disorders for data analysis (incarcerated participants). Over 80% of incarcerated participants met criteria for a past substance use disorder, accounting for the majority of past Axis I disorders in the sample (see Table S1 for a breakdown of SCID diagnoses).

Drug use history was collected using a modified version of the Addiction Severity Index (ASI; Mclellan et al., 1992). This assessment asked participants about their past use of alcohol, heroin, other opiates, cocaine, cannabis, methamphetamine, other amphetamines, and nicotine. For each of these substances, participants were asked if they ever used that substance three or more times per week and for how long they used the substance at least that often. To compensate for age as a confounding variable, severity of use for each substance was calculated by dividing the number of months of regular use by the age of the participant at the time of the interview. Abstinence information was collected in two different ways (see Supplementary Materials for additional information). Seventy-seven of the 306 incarcerated participants were simply asked how much of the substance in question they had used within the past 30 days. Those who reported no use were coded as “abstinent” for that substance. The remaining 229 incarcerated participants were asked to give an approximate date for the last time they had used the substance in question. For this sample, duration of abstinence was set by calculating the number of months between their last reported use and the date at which the assessment was administered (see Table S2 for additional substance use information).

The Balanced Inventory of Desired Responding (BIDR) was administered in order to gauge the accuracy of other self-report measures. The BIDR (Paulhus, 1984) is a 40 item inventory using a 7-point Likert scale consisting of two subscales: Self-Deception and Impression Management. The Self Deception scale is designed to assess defensiveness toward personal threats and positively biased responding that the respondent believes to be true (e.g., “I am a completely rational person”). The Impression Management scale is designed to measure responding that is guided by a desire to create a favorable impression on others (e.g., “I never take things that don't belong to me”).

IQ was estimated using the Vocabulary and Matrix Reasoning subtests of the Wechsler Adult Intelligence Scale (WAIS; Wechsler, 1997; Ryan et al., 1999). Written informed consent was obtained from all participants, after a complete description of the study procedures. The study was reviewed and approved by the University of New Mexico Human Research Review Committee. Participants received monetary compensation for participation. Incarcerated participants were paid at a rate commensurate to work assignments at their facility.

### Stimuli and task

Three sets of pictures (25 moral, 25 non-moral, 25 neutral) were selected, largely from the International Affective Picture System (IAPS; Lang et al., 1995), and supplemented with pictures from media sources. Each moral picture depicted an unpleasant social scene which included a moral violation (e.g., one person attacking another). Non-moral pictures also showed unpleasant social scenes, but they did not contain moral content (e.g., two individuals arguing). Neutral pictures depicted social scenes which were both affectively neutral and devoid of moral content (e.g., two individuals having a conversation). Moral and non-moral pictures were selected based on ratings from a pilot study in which participants rated 100 unpleasant pictures on the moral status of the action depicted. Pictures which were rated highest and lowest on moral violation severity were assigned to the moral and non-moral conditions, respectively. Moral and non-moral pictures were also matched on emotional arousal and social complexity, and neutral pictures were matched to moral and non-moral pictures for social complexity. Within studies in which moral violation severity ratings were collected, the pictures in the moral subset have consistently been rated significantly higher on moral violation severity than non-moral pictures (Harenski and Hamann, 2006; Harenski et al., 2010b).

Participants were told that they would see a series of pictures depicting various people and events. For each picture, they were instructed to determine whether the social scene shown was taking place indoors or outdoors. Participants were not informed of the moral/non-moral distinction across pictures. After being given these instructions, participants completed at least five practice trials in the scanner to ensure that they understood how to perform the task. For each trial, a picture from one of the three sets was displayed on screen for 6 s. Then, participants were presented with a screen reading “Indoor/Outdoor?” and given 4 s to indicate their answer. They were instructed to press one button with their index finger if the picture occurred indoors, and a different button with their middle finger if the picture occurred outdoors. Each of the three picture conditions contained the same proportion of indoor and outdoor pictures (44/56%).

Moral, non-moral, and neutral picture trials were presented in a random order and interspersed with 25 null trials in which a fixation cross was presented for the entire trial. The 100 total trials were presented across two separate runs of the task. Images were rear-projected into the scanner using an LCD projector, controlled by a PC computer. Tasks were designed and presented and responses were recorded using Presentation software (Version 10.78, http://www.neurobs.com/).

### MRI data acquisition and analysis

MR images were collected using a mobile Siemens 1.5T Avanto with advanced SQ gradients (max slew rate 200 T/m/s, 346 T/m/s vector summation, rise time 200 μs) equipped with a 12-element head coil. The EPI gradient-echo pulse sequence (TR/TE 2000/39 ms, flip angle 90°, FOV 24 × 24 cm, 64 × 64 matrix, 3.4 × 3.4 mm in-plane resolution, 5 mm slice thickness, 30 slices) effectively covered the entire brain (150 mm) in 2.0 s. Head motion was minimized using padding and restraint.

To correct residual head motion, “bad” images (confounded by motion or radio-frequency spikes) were estimated and removed using ART-Repair (Mazaika et al., 2009). These images were determined by calculating the mean intensity for a given time series and identifying individual images whose intensity was greater than four standard deviations from the mean. The offending image(s) were replaced in the time series by a rolling mean image, and regressed in the statistical model. The mean number of images removed across participants was 1.98 (of 356).

Imaging data were analyzed using SPM5 (www.fil.ion.ucl.ac.uk/spm/software/spm5). Functional images were spatially normalized to the MNI template and smoothed (8 mm FWHM). Picture presentations (moral, non-moral, neutral) and the rating period for all pictures were modeled as four separate events. Each event was modeled with a six (picture) or four (indoor/outdoor judgment) second hemodynamic response function. Functional images were computed for each participant that represented brain activation associated with viewing moral, non-moral, or neutral pictures. The moral > non-moral picture comparison evaluated brain activation to morally-salient pictures while controlling for general emotional and social content.

The relationship between substance use and neural activity can be characterized by both the general effect of substance use, and by correlations between the severity of substance use and neural activity. Moreover, substance use disorders are generally conceptualized as taxa (DSM-5; ICD-10), although dimensional models have been proposed more recently (e.g., Muthén, 2006; Eaton et al., 2015; for review see Haslam et al., 2012). Consequently, the relationship between hemodynamic responses during the moral > non-moral condition and cocaine use was analyzed in two stages. First, a two-sample *t*-test compared the CU group to the mNCU group. This comparison group was matched on age, IQ, psychopathy and severity of use and duration of abstinence for the following substances: alcohol, cannabis, heroin, other opiates, other amphetamines and nicotine. Second, severity of cocaine use was entered into a regression with individual moral > non-moral contrast images only for CUs. Age, IQ, psychopathy and severity of use for alcohol, cannabis, heroin, other opiates, other amphetamines, and nicotine were also entered in as covariates into this regression. Only CUs were included in this regression analysis because severity of cocaine use was positively skewed within the full incarcerated sample (skewness = 1.946, kurtosis = 2.898). To correct for skew within the CU sample, severity of cocaine use was calculated by taking the square root of the duration of regular use divided by the participant's age (Ermer et al., 2012).

Additionally, duration of abstinence from regular cocaine use was entered into a regression with individual moral > non-moral contrast images only for CUs who had abstinence information available (*n* = 61). Age, IQ, psychopathy, severity of use for cocaine, alcohol, cannabis, heroin, other opiates, other amphetamines and nicotine, and duration of abstinence for alcohol, cannabis, heroin, other opiates, other amphetamines and nicotine were also entered in as covariates into this regression (see Supplementary Materials for additional information). There were no significant group differences between CUs, mNCUs, or NCUs in the number of abstinent participants or the duration of abstinence for any substances (see Tables S2A–C).

Age and IQ scores were not significantly correlated with PCL-R or Factor scores. Consistent with the extant literature, CUs were significantly higher on Factor 2 scores, but not on Factor 1, compared to the NCU sample (see Table 2). IQ was not significantly correlated with any cocaine use measures.

Analyses were performed on a voxel-by-voxel basis over the entire brain using the general linear model in SPM. Hypotheses were tested in regions of interest (PCC, vmPFC, ACC, vSTR, dlPFC). Peak coordinates for these regions were drawn from the moral > non-moral functional maps of a separate sample of 36 non-incarcerated participants (see Table 3 for results). In all a priori regions of interest, family-wise error extent thresholds were small-volume corrected using 10 mm spheres surrounding the peak coordinate.

**Table 3:** **Imaging results for healthy controls and full incarcerated sample**.

**Region**	***BA***	***x***	***y***	***z***	***t***	***k***	***p***	***x***	***y***	***Z***	***t***	***k***	***p***
L. TPJ	39	−48	−60	30	5.05	594	0.000	−51	−60	27	13.53	7810	0.000
R. TPJ	39	54	−48	24	4.55	213	0.004	54	−54	24	10.14		
L. Fusiform	19/37	−21	−45	−9	3.65	29	0.95	−24	−42	−12	13.19		
L. MTG	21	−48	−18	−18	3.49	16	0.998	−57	−21	−12	8.09		
L. MTG	21	−51	−33	−3	3.1	6	1	−60	−36	−6	7.02		
B. PCC	23/31	6	−60	30	4.8	236	0.002	0	−18	39	3.32	4	0.979
L. dmPFC	9	−9	60	33	4.13	118	0.076	−3	48	36	9.39	3063	0.000
L. rostral ACC	32	15	39	15	3.22	2	1	−6	51	15	8.84		
L. dlPFC	8	−42	15	54	3.91	24	0.981	−48	15	45	8.4		
R. dlPFC	8	24	27	54	3.35	12	1	45	15	45	5.5	149	0.001
L. vmPFC	47	−33	21	−21	3.79	22	0.988	−30	21	−15	4.95		
L. vmPFC	10	−21	60	21	2.8	1	1	−24	57	27	5.08		
R. vmPFC	10	3	57	−9	2.77	1	1	−3	57	−9	6.42		
L. vlPFC	45/44	−54	21	18	3.62	12	1	−57	21	15	5.91		
R. vlPFC	47							42	30	−12	3.72	9	0.879
L. ventral ACC	32	−12	45	0	3.39	8	1	−30	60	12	3.36	5	0.965
L. Putamen	49	−21	9	9	3.3	17	0.998	−12	3	9	3.28	2	0.995
L. Temporal Pole	38	−51	6	−33	2.98	2	1	−39	18	−33	3.19	1	0.998
R. Cerebellum	NA	18	−72	−30	2.25	10	1	24	−81	−33	6.25	375	0.000
R. Thalamus	50	18	−15	0	2.65	19	1	15	−21	9	3.89	4	0.979

*BA, Brodmann area; t, t score; k, spatial extent of activation; p, corrected p-value. Activations are reported in MNI coordinate space. Areas in which the Incarcerated sample showed greater activation than Healthy Controls are highlighted in red. Areas in which the Healthy Controls showed greater activation than the Incarcerated sample are highlighted in green. TPJ, Temporoparietal Junction; MTG, Middle Temporal Gyrus; PCC, Posterior Cingulate Cortex; dmPFC, Dorsomedial Prefrontal Cortex; ACC, Anterior Cingulate Cortex; dlPFC, Dorsolateral Prefrontal Cortex; vmPFC, Ventromedial Prefrontal Cortex; vlPFC, Ventrolateral Prefrontal Cortex*.

Whole-brain analyses were thresholded at *p* < 0.001, and FWE corrected using cluster-extent based thresholding with primary threshold of *p* < 0.05. Cluster thresholds for whole-brain family-wise error multiple comparison correction were derived from Monte Carlo simulations (3dClustSim, AFNI, http://afni.nimh.nih.gov). For the group comparison of CUs and mNCUs, the cluster threshold was set at ≥ 1026 mm^3^ (38 contiguous voxels). For the regression analysis within the entire incarcerated sample, the cluster threshold was set more stringently at ≥ 1134 mm^3^ (42 contiguous voxels).

### Imaging results

**Figure 1:**
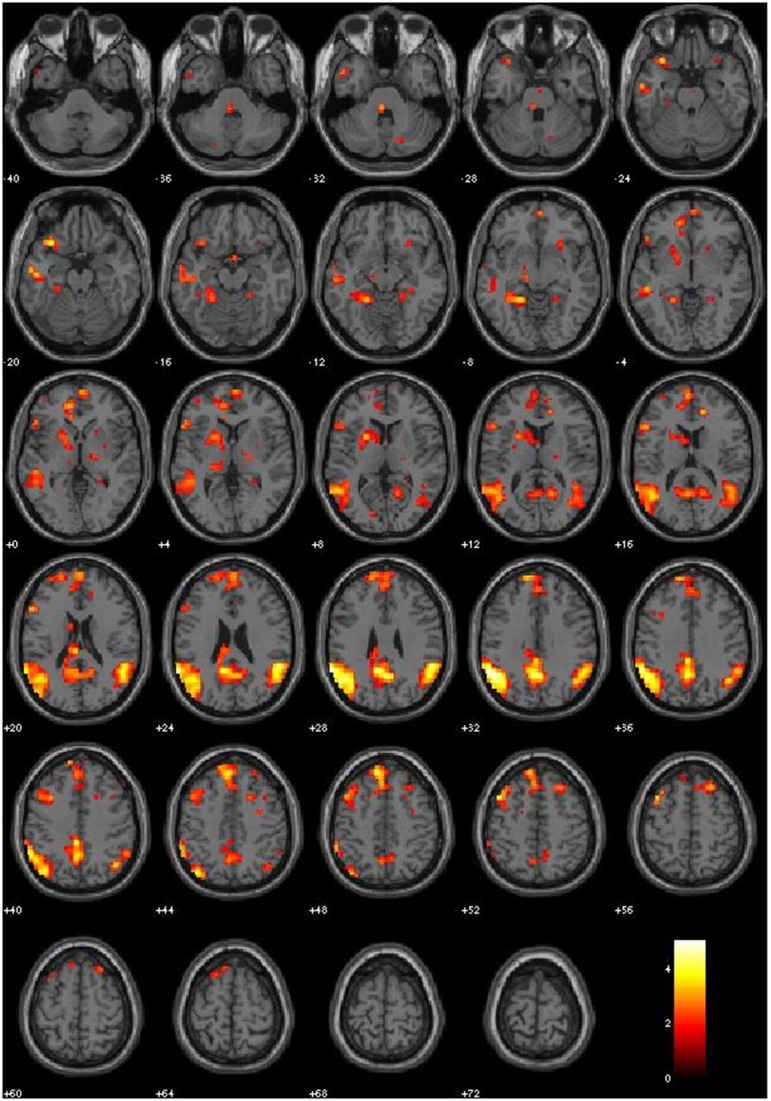
**Main Effect of Moral > Non-Moral condition in healthy controls (p-threshold = 0.05)**.

We first examined the main effects of viewing moral > non-moral pictures across all non-incarcerated participants (Figure 1). Consistent with a previous study employing this task in a non-clinical population, the main effect of moral vs. non-moral pictures in the non-incarcerated sample revealed increased hemodynamic response in regions previously implicated in moral intuition (Harenski et al., 2010a)— including the bilateral temporo-parietal junction and posterior cingulate cortex. The main effect of moral vs. non-moral pictures in the non-incarcerated sample showed a similar pattern of results (see Table 3 for main effects).

Relative to matched non-cocaine users, cocaine users showed significantly reduced hemodynamic activity in the moral > non-moral contrast in several cortical and subcortical regions. Whole-brain analysis revealed reduced activity in both the left subgenual anterior cingulate cortex (sgACC) and right superior frontal gyrus (SFG). Small-volume correction revealed additional areas of hypoactivation. In the left hemisphere, these included the ventral and dorsal striatum (vSTR and dSTR), ventral anterior cingulate cortex (vACC), ventromedial prefrontal cortex (vmPFC), and middle temporal gyrus (MTG). On the right, hypoactivity was observed in the dorsomedial prefrontal cortex (dmPFC) extending into the rostral anterior cingulate cortex (rACC), and ventrolateral prefrontal cortex (vlPFC) extending into the anterior insula (aINS). Hypoactivation was observed bilaterally in the parahippocampal gyrus (PHG) and anterior thalamus (THAL). Cocaine users did not show greater hemodynamic activity in the moral > non-moral contrast, compared to the mNCUs, in any regions of interest (see Table 4).

**Table 4:** **Imaging results from group comparison of Cocaine Users (CU) and Matched Non-Cocaine Users (mNCU)**.

**Moral** > **Non-Moral**							
***mNCU** > **CU***	***BA***	***x***	***y***	***z***	***t***	***k***	***p***
L. subgenual ACC	32/25	−6	21	−3	4.62	52	0.056
R. Superior Frontal Gyrus	10	24	48	12	4.88	39	0.134
R. Middle Frontal Gyrus^*^	10/32	21	45	12	4.04	64	0.004
R. Inferior Frontal Gyrus^*^	47	33	24	−18	3.25	33	0.044
L. Medial Frontal Gyrus^*^	10	−3	54	−6	3.32	64	0.037
L. ventral ACC^*^	10/11/32	−9	42	−6	4.05	98	0.004
R. Cuneus^*^	19/18/17	12	−63	6	3.66	124	0.014
R. Precuneus^*^	7	15	−75	54	3.7	145	0.012
R. Parahippocampal Gyrus^*^	19/36/37	21	−45	−6	3.51	77	0.022
L. Middle Temporal Gyrus^*^	21/37	−48	−48	−3	3.32	86	0.037
L. Parahippocampal Gyrus^*^	36	−18	−42	0	3.62	41	0.016
L. Thalamus^*^	50	−9	−3	6	3.26	81	0.043
R. Thalamus^*^	50/48	12	−6	15	3.13	15	0.059
***CU** > **mNCU***							
None							

BA, Brodmann area; t, t score; k, spatial extent of activation. Activations are reported in MNI coordinate space. Regions marked with an asterisk (^*^) were identified after small volume correction using regions of interest; p-values for these regions represent FWE small-volume-corrected values. Regions without an asterisk were identified during whole-brain analysis; p-values for these regions are corrected p-values.

Within the CU sample, no significant correlations were found between hemodynamic activity in the moral > non-moral contrast and severity of cocaine use. Factor 1 scores, however, were negatively correlated with hemodynamic activity in the right vSTR and rACC in the moral > non-moral contrast (see Table 5). For those in the CU sample with duration of abstinence information (*n* = 61), duration of abstinence from cocaine was negatively correlated with hemodynamic activity in the right posterior cingulate cortex (PCC) for the moral > non-moral contrast (see Table 6).

**Table 5:** **Imaging results from regression with psychopathy within the cocaine users group**.

**Moral > Non-Moral**							
**Factor 1**	***BA***	***x***	***y***	***z***	***t***	***k***	***p***
***Positive Correlation***							
None							
***Negative Correlation***							
R. ventral Striatum	52/48	9	3	−9	3.82	17	0.011
R. rostral Anterior Cingulate Cortex	32	15	33	21	3.47	18	0.031
**Factor 2**							
None							

BA, Brodmann area; t, t score; k, spatial extent of activation. Activations are reported in MNI coordinate space. Regions marked with an asterisk (^*^) were identified after small volume correction using regions of interest; p-values for these regions represent FWE small-volume-corrected values. Regions without an asterisk were identified during whole-brain analysis; p-values for these regions are corrected p-values.

**Table 6:** **Imaging results from regression with duration of abstinence from cocaine within the cocaine users group**.

**Moral > Non-Moral**							
**Cocaine Abstinence**	***BA***	***x***	***y***	***z***	***t***	***k***	***p***
***Positive Correlation***							
None							
***Negative Correlation***							
R. posterior Cingulate Cortex^*^	23	18	−60	2	3.6	102	0.026

BA, Brodmann area; t, t score; k, spatial extent of activation. Activations are reported in MNI coordinate space. Regions marked with an asterisk (^*^) were identified after small volume correction using regions of interest; p-values for these regions represent FWE small-volume-corrected values. Regions without an asterisk were identified during whole-brain analysis; p-values for these regions are corrected p-values.

## Discussion

The present study investigated whether cocaine abuse held any relationship to the neural systems underlying moral intuition, and what impact psychopathic traits might have on this relationship. Consistent with our hypothesis, individuals in the Cocaine Users group exhibited significantly reduced activation while viewing moral pictures relative to non-moral pictures in several cortico-limbic regions, compared to their non-cocaine using counterparts. In the left hemisphere, these included the ventral striatum (vSTR), subgenual cingulate cortex (sgACC), and ventromedial prefrontal cortex (vmPFC). On the right, hypoactivity was observed in the dorsomedial prefrontal cortex (dmPFC) extending into the pregenual anterior cingulate cortex (pgACC), and ventrolateral prefrontal cortex (vlPFC) extending into the anterior insula (aINS). Additionally, cocaine users higher in psychopathic traits exhibited additional neural abnormalities in the right vSTR and more severe aberration in the right pgACC.

CUs exhibited reduced activity in the left vSTR during moral vs. non-moral picture processing, when compared to mNCUs. Moreover, within the CU group, hemodynamic activity in the right vSTR was negatively correlated with Factor 1 scores. A plethora of research has established that this reward pathway is involved in the perception of social stimuli and social interaction (Skuse and Gallagher, 2009; Volkow et al., 2011; Lin et al., 2012), and is theorized to be crucially involved in motivating prosocial behavior (Moll et al., 2006; Izuma et al., 2010). During moral intuition, the vSTR may be partially responsible for the generation of a negative affective state associated with witnessing an immoral act. Although, commonly associated with positive feedback linked to reward, the vSTR has also been implicated in the generation of negative emotional states (Horvitz, 2000; Pruessner et al., 2004).

Functional abnormalities within the vSTR are consistent with our etiological account of cocaine's effect on social cognition. Acute administration of cocaine increases extracellular dopamine (DA) levels by blocking presynaptic dopamine transporter (DAT; Ritz et al., 1987). With regular use, compensatory changes begin to occur, such as downregulation of DA receptors, transporters, and function (Adinoff, 2004). Cocaine abusers have shown reductions in D2 receptor binding, extending into prolonged abstinence (Volkow et al., 1990), which may be associated with increased DA transporter availability (Bowers et al., 1998). One of the most common functional abnormalities exhibited by cocaine users is and over-reactivity to cocaine-related stimuli and under-reactivity to stimuli with any other contant (Aguilar de Arcos et al., 2005; Volkow et al., 2010), and this differential reactivity is generally associated with aberrant vSTR activity (Volkow et al., 2006; Childress et al., 2008; Asensio et al., 2010; Dunning et al., 2011; Holroyd et al., 2014). Our findings suggest that, in addition to being under-reactive to non-drug rewards, cocaine users may also be under-reactive to aversive stimuli which are unrelated to cocaine use, such as depictions of immoral acts.

As part of the mesocorticolimbic domapine pathway, the vSTR projects to many of the other brain regions in which CUs exhibited hypoactivity— including the mPFC, ACC and aINS (Haber, 2011). These areas also work collectively as part of the “visceromotor network”— which is thought to play a critical role in the identification of the emotional significance of environmental stimuli, the production of affective states, and automatic regulation of these autonomic responses to emotive stimuli via the production of visceral states (Phillips et al., 2003; Critchley, 2005; Lindquist et al., 2012). During social cognition, this network is largely responsible for our tendency to reflexively embody the bodily states of observed agents— a process thought to be a central component of affective empathy (Decety and Jackson, 2004; Lamm et al., 2011; Zaki and Ochsner, 2012; Raz et al., 2014) and social cognition more generally (Vogt, 2005; Amodio and Frith, 2006).

Both moral and non-moral pictures have negatively valenced affective content insofar as they depict people who are suffering. Moral pictures, however, are distinct in that they depict a transgressor who is inflicting that suffering on a victim. This additional feature is likely to give moral stimuli additional affective salience— stemming from a reflexive appreciation of the affective state of the victim or an aversive response to the “wrongness” of the transgressor's behavior. Hypoactivity within this visceromotor network during moral vs. non-moral picture processing suggests that CUs are relatively insensitive to this difference in affective content. In other words, the neural abnormalities identified may be indicative of an aberration in the affective component of moral intuition in cocaine users.

In contrast to mNCUs, who recruited the left vmPFC more during moral compared to non-moral picture processing, CUs exhibited similar hemodynamic activity in this region across both types of stimuli. The vmPFC has been implicated in moral cognition across a myriad of studies (e.g., Greene et al., 2001; Moll et al., 2001, 2002a,b; Heekeren et al., 2003; Harenski and Hamann, 2006; Luo et al., 2006; Schiach-Borg et al., 2006; Prehn et al., 2007; Harenski et al., 2009; Young and Saxe, 2009; Shenhav and Greene, 2010; Sommer et al., 2010). Within healthy populations, the vmPFC is responsible for integrating emotional information into decision-making (Rolls, 2004; Bechara and Damasio, 2005; Lindquist et al., 2012) and is therefore thought to be involved in the affective aspects of moral cognition (Greene et al., 2001; Moll et al., 2002a,b; Heekeren et al., 2003). Individuals who have suffered damage to this area often exhibit impulsive and callous behavior (Rolls et al., 1994; Grafman et al., 1996; Brower and Price, 2001) along with abnormal moral cognition, which is generally interpreted to be the result of an emotional deficit (Ciaramelli et al., 2007; Koenigs et al., 2007; Moretto et al., 2010; Thomas et al., 2011). Increased activity during moral picture processing in the vmPFC may therefore be the result of a stronger affective reaction to the moral content of those stimuli. Correspondingly, similar recruitment of this region across both types of stimuli may be the result of a comparatively reduced affective response to moral stimuli.

Cocaine users showed a similar abnormal pattern of neural activity in two regions of the pgACC—one adjacent to the dmPFC (rACC) and the other adjacent to the vmPFC (vACC). In both these regions, mNCUs exhibited greater hemodynamic activity for moral vs. non-moral stimuli, while CUs did not. The pgACC is selectively engaged when task-irrelevant information interferes with performance due to its emotional content (Whalen et al., 1998; Haas et al., 2006; Mohanty et al., 2007; Egner et al., 2008; Ochsner et al., 2009; Etkin et al., 2011; Kanske and Kotz, 2011a,b; Iordan et al., 2013). The more dorsal portion of the pgACC helps resolve these emotional conflicts by inhibiting incongruent emotional reactions (Ochsner et al., 2004; Koenigs and Tranel, 2007; Diekhof et al., 2011; Amemori and Graybiel, 2012), possibly through a down-regulation of amygdalar activity (Etkin et al., 2006; Bissière et al., 2008). Importantly, however, the need for regulation does not appear to be necessary for pgACC activity. Several studies have found ACC activity in response to emotional stimuli even in contexts where attentional control was not required for performance (Pissiota et al., 2003; Kosson et al., 2006; Nakic et al., 2006; Chiu et al., 2008; Todd et al., 2014). It is likely, therefore, the pgACC is involved both in the identification of salient emotional information and the emotional conflict regulation.

Compared to mNCUs, CUs exhibited substantially less hemodynamic activity in the sgACC during moral relative to non-moral picture processing. Functionally, the sgACC has been consistently implicated in emotional processing (Cromheeke and Mueller, 2014). This area has strong projections to visceral and emotional control centers such as the amygdala and ventral striatum (Johansen-Berg et al., 2007; Vogt and Vogt, 2009) and is thought to play a crucial role in mood disorders marked by anhedonia (Greicius et al., 2007; Drevets et al., 2008). Recent research has also implicated the sgACC in socio-moral emotions such as guilt, a self-referential emotion generally elicited by the violation of moral norms (Zahn et al., 2009a; Green et al., 2010; Basile et al., 2011; Wagner et al., 2011), and empathic concern for the victims of harmful actions (Zahn et al., 2009b; Decety et al., 2012; Wiech et al., 2013). sgACC activity has also been implicated in the implicit processing of morally salient statements (Luo et al., 2006; Schiach-Borg et al., 2008).

**Figure 2:**
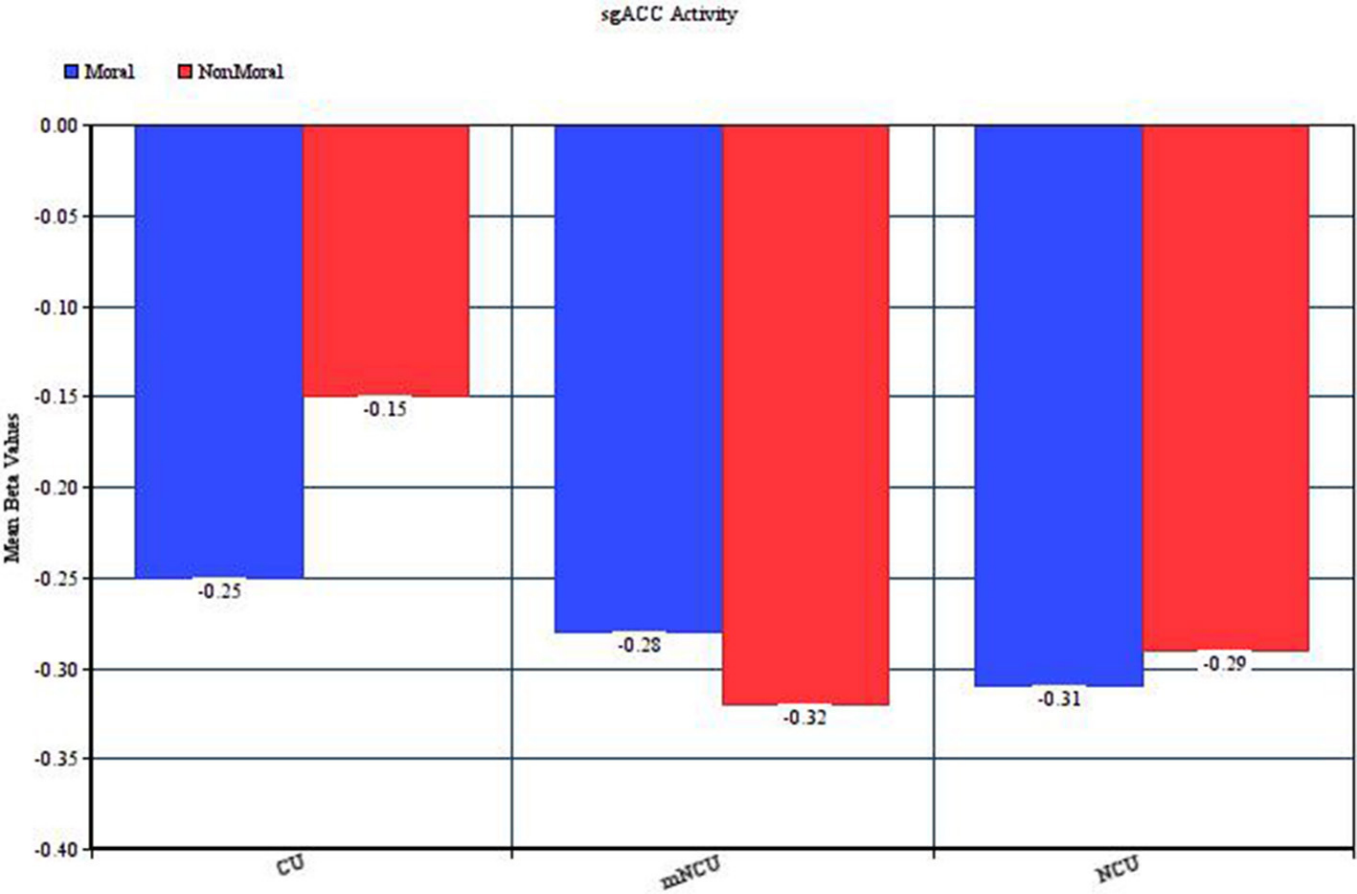
**Activity in the left subgenual ACC during the Moral > Non-Moral condition**.

One noteworthy complication in this interpretation is that, unlike all the other regions of hypoactivity amongst the CUs, the pattern of results in the sgACC indicated similar deactivation in the moral and non-moral conditions for the NCUs and greater deactivation in the moral relative to non-moral condition for the cocaine users (see Figure 2). Given the purported role of the sgACC in empathic concern and the fact that both moral and non-moral stimuli depicted suffering, similar activation in this area between stimulus types might be expected. Conversely, however, this would imply that the cocaine users had less of an empathic response when the suffering was due to an immoral act than when it was not. Research on the role of the sgACC in social cognition, especially moral processing, is in its infancy. Future investigations may find that this region plays a much more complex part in moral cognition, and help to provide an explanation for this finding.

Lastly, it is worth noting that, within the Cocaine User group, no significant correlations were found between the severity of cocaine use and hemodynamic activity during moral picture processing. Many functional neuroimaging studies on substance use have found similar results. This discrepancy might be resolved with an improved measure for cocaine use severity. Additional information which may improve severity measures includes: the age(s) of the participant during regular use, the average quantity of cocaine used during periods of regular use and the duration of abstinence from cocaine use prior to scanning. Moreover, devising a substance use measure which applies to individuals who used a drug with some frequency, but fewer than three times a week, would help to normalize the distribution of that measure within the population.

Consistent with our second hypothesis regarding the interaction between psychopathy and cocaine abuse, within the cocaine users, higher Factor 1 scores were associated with reduced activity in the right vSTR and rACC. These regions have been implicated separately in both substance abuse and psychopathy research

Several studies have identified functional (Kiehl et al., 2001; Buckholtz et al., 2010; Bjork et al., 2012; Decety et al., 2013; Pujara et al., 2013) and structural (Glenn et al., 2010; Schiffer et al., 2011; Boccardi et al., 2013; Hoppenbrouwers et al., 2013) striatal abnormalities in individuals high in psychopathic traits. Individuals who are less empathic would be expected to have less of a negative affective reaction to witnessing someone else being wronged. Reduced vSTR activity while observing immoral acts in cocaine users who exhibit more of the interpersonal and affective psychopathic traits, is consistent with the purported role of this region in moral intuition.

Similarly, both cocaine users and psychopaths have demonstrated hypoactivity in the right rACC. Within psychopathy research, hypoactivity within the rACC has been associated with reduced allocation of attention toward the affective content of stimuli (Kiehl et al., 2001; Müller et al., 2003; Birbaumer et al., 2005). Several different etiological models of psychopathy have proposed an attention deficit as being central to the disorder (MacCoon et al., 2004; Hiatt and Newman, 2006; Kiehl, 2006). The paralimbic dysfunction model hypothesizes that the deficit is in emotional attention specifically—whereby psychopaths are under-reactive to affective information outside the current focus of attention (Kiehl, 2006). This hypothesis has been supported by a number of studies showing that psychopaths tend to perform better than average when the task containing emotional information that is incongruent with performance (Christianson et al., 1996; Mitchell et al., 2006) and worse when it is relevant (Williamson et al., 1991; Lorenz and Newman, 2002). However, the fact that this region was also found to be hypoactive in CUs compared to mNCU, who did not differ on Factor 1 scores suggests that cocaine use has an independent and potentially additive impact on attentional allocation during implicit moral processing. Within the substance abuse literature, abnormal neural activity in the rACC amongst cocaine users has primarily been associated with the ‘attentional bias’ they exhibit toward cocaine-related stimuli (Goldstein et al., 2007, 2009, 2010; Ersche et al., 2010). An attentional bias toward cocaine related stimuli may have resulted in an attentional bias away from the morally relevant affective content of the stimuli. It is important to note, however, that support for this interpretation is limited by the fact that participants' allocation of attention during the implicit moral task was not directly measured. Future research into the interaction between substance abuse and psychopathy may benefit from testing this interpretation more rigorously. During implicit moral processing, this bias appears to have resulted in cocaine users being insensitive to the morally relevant affective content in the stimuli. Similarly, several different etiological models of psychopathy have proposed an attention deficit as being central to the disorder (Kiehl, 2006; Hiatt and Newman, 2006; MacCoon et al., 2004). The paralimbic dysfunction model hypothesizes that the deficit is in emotional attention specifically—whereby psychopaths are under-reactive to affective information outside the current focus of attention (Kiehl, 2006). This hypothesis has been supported by a number of studies showing that psychopaths tend to perform better than average when the task containing emotional information that is incongruent with performance (Christianson et al., 1996; Mitchell et al., 2006) and worse when it is relevant (Lorenz and Newman, 2002; Williamson et al., 1991). Moroever, this under-reactivity to affective information has been associated with hypoactivity in the rACC amongst psychopaths (Kiehl et al., 2001; Müller et al., 2003; Birbaumer et al., 2005).

In summary, former regular cocaine users exhibited abnormal neural activity during implicit moral cognition in several corticolimbic brain regions. Unlike their non-cocaine using peers, members of the Cocaine Users group failed to discriminate between morally irrelevant depictions of negative social scenarios and depictions of moral violations at a neural level. The frontostriatal regions in which this abnormal hypoactivity occurred suggest that this reflexive, perceptual discrimination is most likely rooted in affective processes. Thus, while the majority of the sample found the moral pictures to be more affectively arousing than the non-moral ones, those who had once regularly used cocaine did not.

These findings add to a growing body of research suggesting a deleterious effect of substance abuse on high-level socio-affective processes like moral cognition. Not only can drugs of abuse alter our natural reward pathway so that one does not find non-drug rewards to be arousing, but it can also lead to insensitivity toward negative stimuli— such as witnessing an immoral act. Consequently, abnormalities in this pathway, such as those seen in chronic cocaine abusers, may correspond to significant alterations in individuals' social cognitive processing. Within moral psychology, this is consistent with an increasingly popular view that striatal reward processing plays an important role in moral cognition— both in promoting prosociality and depressing immoral or antisocial behaviors. Within addiction research, alterations in socio-affective processing may help to explain both the antisocial behaviors of current and former addicts and the propensity of former addicts to relapse into substance use many years after withdrawal. Overvaluation of the individual's drug of choice may be coupled with a devaluation of prosocial reinforcers, such as spending time with friends or family, and a reduced aversion toward the negative outcomes associated with substance abuse.

## Limitations

Several limitations of this study should be noted. First, the results of this study cannot be used to make any strong claims regarding the casual influence of cocaine use on moral intuition. It is possible that individuals prone to regular cocaine use exhibited the observed deficits in moral intuition before they began using cocaine.

Second, substance use severity ratings were based on self-report, allowing for the possibility that some participants under-reported their past or current substance use. However, there are several reasons to assume that actual under-reporting within our population was relatively low. Current substance use may have been under-reported due to a fear of negative consequences for reporting current drug use. However, all participants were assured that all of the information they revealed about themselves would remain confidential and that none of what they reported to the interviewer would have any impact on their institutional status. Additionally, past or current substance abuse may have been under-reported due to social desirability concerns. Existing research on the accuracy of self-reported cocaine use within incarcerated populations suggests that, while under-reporting does occur, incarcerated participants are less likely to under-report due to social desirability concerns than community samples (Harrison and Hughes, 1997; Hser, 1997; Fendrich et al., 1999). Within our own population, CUs and mNCUs did not significantly differ on impression management or self-deception, as measured by the BIDR. Moreover, within the CUs, social desirability was not correlated with self-reported cocaine use or self-reported abstinence from cocaine use.

The present study used a 1.5T Mobile MRI scanner. Whereas, we have published on the reliability of 1.5T for fMRI (Kiehl et al., 2005), it is possible that additional effects may be found at higher field strengths. Nevertheless, we were able to confirm our hypotheses (and collect the data from this prison sample) with 1.5T MRI.

Finally, based on data for a separate study where urinary toxicology screens were administered during a research study at the same site, stimulant use during incarceration appears to be relatively uncommon and under-reporting of current stimulant use appears to be very low. Of 62 incarcerated participants taking part in a substance abuse treatment program, only 2 reported stimulant use during their incarceration. Additionally, out of 34 incarcerated participants who completed the ASI and received a UA, only 1 participant reported no current cocaine use, but tested positive for cocaine (Kiehl laboratory, unpublished data).

### Conflict of interest statement

The authors declare that the research was conducted in the absence of any commercial or financial relationships that could be construed as a potential conflict of interest.
