# Supplementary material for: Abnormal frontostriatal activity in recently abstinent cocaine users during implicit moral processing
Source: Front Hum Neurosci. 2015 Oct 16;9:565. doi: 10.3389/fnhum.2015.00565 (PMC4608360; doi:10.3389/fnhum.2015.00565)
Supplement: Supplementary file 1 [file Table1.DOCX]

**Table S1a: Breakdown of Axis I SCID Diagnoses for Incarcerated Sample (n=298)^[[1]](#footnote-1)^**

| **Past Disorder** | **%** | |
| --- | --- | --- |
| *Mood Disorders* | 6.53 | |
| Biploar | 0.33 | |
| Major Depression | 4.25 | |
| Dysthymic Disorder | 0.33 | |
| Depressive Disorder NOS | 0.33 | |
| Substance-Induced | 1.31 | |
| *Psychotic Disorders* | 0.33 | |
| Psychotic Disorder NOS | 0.33 | |
| *Substance Use Disorders* | *Dependence* | *Abuse* |
|  | 54.7 | 65.1 |
| Alcohol | 31.54 | 32.89 |
| Sedative-Hypnotic-Anxiolytic | 4.7 | 5.37 |
| Cannabis | 25.5 | 37.92 |
| Stimulants | 13.09 | 7.72 |
| Opiods | 15.77 | 5.7 |
| Cocaine | 20.81 | 11.41 |
| Hallucinogens/PCP | 3.36 | 10.07 |
| Polysubstance | 2.68 | 0 |
| Other | 1.01 | 3.36 |
| *Anxiety Disorders* | 6.71 | |
| Panic | 2.35 | |
| Phobia | 2.36 | |
| Obsessive-Compulsive | 0.34 | |
| Post-Traumatic Stress | 1.34 | |
| Generalized Anxiety | 0.34 | |
| Anxiety Disorder NOS | 0.67 | |

**Table S1b: Breakdown of Axis II SCID Diagnoses for Incarcerated Sample (n=298)^[[2]](#footnote-2)^**

| **Axis II Disorder** | **%** |
| --- | --- |
| Avoidant Personality | 1.03 |
| Dependent Personality | 0 |
| Obsessive-Compulsive Personality | 1.72 |
| Paranoid Personality | 2.41 |
| Schizotypal Personality | 0.34 |
| Schizoid Personality | 1.03 |
| Narcissistic Personality | 0 |
| Borderline Personality | 2.41 |
| Histrionic Personality | 2.07 |
| Antisocial Personality | 50 |

1. SCID data was missing for 8 Incarcerated participants. [↑](#footnote-ref-1)
2. SCID data was missing for 8 Incarcerated participants. [↑](#footnote-ref-2)
