# Supplementary material for: Abnormal frontostriatal activity in recently abstinent cocaine users during implicit moral processing
Source: Front Hum Neurosci. 2015 Oct 16;9:565. doi: 10.3389/fnhum.2015.00565 (PMC4608360; doi:10.3389/fnhum.2015.00565)
Supplement: Supplementary file 2 [file Table2.DOCX]

***Supplementary Material***

**Abnormal Frontostriatal Activity in Recently Abstinent Cocaine Users During Implicit Moral Processing**

Brendan M. Caldwell^1*^, Carla L. Harenski^1^, Keith A. Harenski^1^, Samantha J. Fede^2^, Michael R. Koenigs^3^, Kent A. Kiehl^1,2^

^1^ The MIND Research Network, Albuquerque, NM 87131, USA

^2^ University of New Mexico, Department of Psychology, Albuquerque, NM 87131, USA

^3^ University of Wisconsin- Madison, Department of Psychiatry, Madison, WI 53706, USA

Correspondence

Brendan M. Caldwell

The MIND Research Network

1101 Yale Blvd NE

Albuquerque, NM, 87131, USA

bcaldwell@mrn.org

**1 Supplementary Methods**

**Measuring Abstinence with the ASI**

Across the entire incarcerated sample, two different modified versions of the Addiction Severity Index (ASI) were administered. The older version, hereafter *ASI v1.0*, was administered to 77 participants. The newer version, hereafter *ASI v2.0*, was administered to the remaining 229. ASI v1.0 asked the following questions for each substance:

1. Have you ever tried ___?

2. How old were you the first time you ever tried ___?

3. Did you ever use ___ on a regular basis?

4. How many years/months did you use ___on a regular basis?

5. If no regular use, how many times did you use or what was your use of ______?

6. How much have you used _____________ in the last 30 days?

7. Route of Administration: How did you use ________________?

In addition to these questions, ASI v2.0 records the following for each substance:

1. The start and end age(s) of regular use- accounting for multiple episodes of use
2. Total number of months of regular use
3. Approximate date of last time substance was used
4. Participant’s Primary Drug of Choice

For participants who received ASI v1.0, the only abstinence information available was whether or not they had used the substance within the past 30 days. For participants who received ASI v2.0, abstinence was calculated as the approximate number of months between the assessment date and their last period of regular substance use (see Table S2a for sample statistics). In order to combine abstinence information for both versions of the ASI, participants in the latter group were also coded based on whether their duration of abstinence for a given substance was longer than 30 days (see Table S2b for full sample statistics).

**MRI Analysis for Duration of Abstinence**

CU and mNCU did not differ significantly in either the number of participants who had used any substance within the past 30 days (see Table S2b) or in the duration of abstinence for any substance other than cocaine (see Table S2c).

Duration of abstinence from cocaine was entered into a regression with severity of cocaine use for CUs only.

**2 Supplementary Tables**

**Table S2a: Duration of Abstinence for Incarcerated Participants receiving ASI v2.0 (n=229)^[[1]](#footnote-2)^**

| **Abstinence from Regular Use ASIX v1.2 only (n=229)** | | | | | |
| --- | --- | --- | --- | --- | --- |
|  | **N (Regular Use)** | **Minimum** | **Maximum** | **Mean** | **Std. Deviation** |
| **Alcohol** | 147 | 1.27 | 334.37 | 97.2787 | 73.34577 |
| **Heroin** | 26 | .77 | 312.47 | 53.8615 | 68.51597 |
| **Cocaine** | 61 | 12.23 | 335.13 | 134.7115 | 86.05677 |
| **Cannabis** | 177 | 2.10 | 382.07 | 89.7896 | 79.83375 |
| **Methamphetamine** | 27 | 4.13 | 291.47 | 92.8654 | 75.16615 |
| **Other Amphetamines** | 20 | 15.10 | 352.47 | 115.5400 | 101.70515 |
| **Nicotine** | 183 | 2.10 | 277.37 | 72.2710 | 61.50774 |
| **Other Opiates** | 42 | 7.33 | 372.33 | 69.5421 | 67.09057 |

**Table S2b: Sample Statistics and Group Comparisons for Substance Use within the Past 30 Days (n=306)**

| **Use in Past 30 Days (n=306)** | | | | | | | |
| --- | --- | --- | --- | --- | --- | --- | --- |
|  |  |  |  |  | **CU vs. mNCU** | | |
|  |  | **CU** | **mNCU** | **NCU** | **χ²** | **df** | **p** |
| **Alcohol** | No | 87 | 87 | 219 | NA |  |  |
|  | Yes | 0 | 0 | 0 |  |  |  |
| **Heroin** | No | 84 | 86 | 218 | 2.024 | 1 | 0.155 |
|  | Yes | 2 | 0 | 0 |  |  |  |
| **Cocaine** | No | 87 | 84 | 214 | NA |  |  |
|  | Yes | 0 | 0 | 0 |  |  |  |
| **Cannabis** | No | 84 | 84 | 213 | 0 | 1 | 1 |
|  | Yes | 2 | 2 | 2 |  |  |  |
| **Methamphetamine** | No | 86 | 85 | 216 | NA |  |  |
|  | Yes | 0 | 0 | 0 |  |  |  |
| **Other Amphetamines** | No | 87 | 87 | 219 | NA |  |  |
|  | Yes | 0 | 0 | 0 |  |  |  |
| **Nicotine** | No | 80 | 83 | 212 | 1.055 | 1 | 0.304 |
|  | Yes | 6 | 3 | 5 |  |  |  |
| **Other Opiates** | No | 83 | 86 | 218 | 0.001 | 1 | 0.98 |
|  | Yes | 1 | 1 | 1 |  |  |  |

**Table S2c: Duration of Abstinence for CUs vs. mNCUs who received ASI v2.0 (n=129)^[[2]](#footnote-3)^**

| **CU vs. mNCU Abstinence (ASI v2.0 only)** | | | | | | | |
| --- | --- | --- | --- | --- | --- | --- | --- |
|  | | **N (Regular Use)** | **Mean** | **Std. Deviation** | **t** | **df** | **p** |
| **Alcohol** | CU | 47 | 103.0355 | 80.74807 | 1.068 | 96 | .288 |
|  | mNCU | 51 | 86.8948 | 68.71945 |  |  |  |
| **Heroin** | CU | 18 | 60.4685 | 80.85184 | .706 | 23 | .487 |
|  | mNCU | 7 | 38.2524 | 24.65439 |  |  |  |
| **Cocaine** | CU | 61 | 134.7115 | 86.05677 |  |  |  |
|  | mNCU | 0 |  |  |  |  |  |
| **Cannabis** | CU | 57 | 91.3678 | 84.79372 | .199 | 114 | .843 |
|  | mNCU | 59 | 88.1847 | 87.42084 |  |  |  |
| **Methamphetamine** | CU | 18 | 75.5630 | 71.61341 | -1.023 | 21 | .318 |
|  | mNCU | 5 | 112.5467 | 70.98896 |  |  |  |
| **Other Amphetamines** | CU | 8 | 143.8250 | 136.00073 | 1.318 | 13 | .210 |
|  | mNCU | 7 | 71.5143 | 52.48659 |  |  |  |
| **Nicotine** | CU | 58 | 68.0356 | 64.17972 | .496 | 115 | .621 |
|  | mNCU | 59 | 62.5938 | 54.20486 |  |  |  |
| **Other Opiates** | CU | 11 | 69.6636 | 52.08619 | -.046 | 23 | .964 |
|  | mNCU | 14 | 70.6595 | 54.69175 |  |  |  |

1. Duration of abstinence was measured in months. Duration of Abstinence = number of months between assessment date and last time substance was used regularly (3 times or more per week). [↑](#footnote-ref-2)
2. Duration of abstinence was measured in months. Duration of Abstinence = number of months between assessment date and last time substance was used regularly (3 times or more per week). CU = Cocaine Users group; mNCU = matched Non-Cocaine Users group. [↑](#footnote-ref-3)
