# Supplementary material for: Abnormal frontostriatal activity in recently abstinent cocaine users during implicit moral processing
Source: Front Hum Neurosci. 2015 Oct 16;9:565. doi: 10.3389/fnhum.2015.00565 (PMC4608360; doi:10.3389/fnhum.2015.00565)
Supplement: Supplementary file 3 [file Table3.DOCX]

**Supplementary Table S3a: Correlations between Psychopathy and Substance Use Duration**

| **PCLR vs. Substance Use History (n=306)** | | | | | | | |
| --- | --- | --- | --- | --- | --- | --- | --- |
|  | **PCLR Total** | **PCLR Factor 1** | **PCLR Factor 2** | **PCLR Facet 1** | **PCLR Facet 2** | **PCLR Facet 3** | **PCLR Facet 4** |
| **Alcohol Use** | -.048 | -.139^*^ | .031 | -.140^*^ | -.134^*^ | -.043 | -.002 |
| **Heroin Use** | .058 | -.036 | .095 | -.077 | -.032 | .031 | .104 |
| **Cocaine Use** | .051 | -.041 | .100 | -.062 | -.058 | .012 | .076 |
| **Methamphetamine Use** | .015 | -.096 | .106 | -.049 | -.112 | .030 | .139^*^ |
| **Cannabis Use** | .129^*^ | -.006 | .204^**^ | -.007 | .010 | .154^**^ | .182^**^ |
| **Other Amphetamine Use** | .044 | -.029 | .087 | -.065 | -.042 | .090 | .067 |
| **Other Opiates Use** | .025 | .006 | .018 | -.076 | .043 | -.008 | .021 |
| **Nicotine Use** | .146^*^ | -.017 | .214^**^ | -.005 | -.031 | .134^*^ | .184^**^ |
| *. Correlation is significant at the 0.05 level (2-tailed). | | | | | | | |
| **. Correlation is significant at the 0.01 level (2-tailed). | | | | | | | |

**Table S3b: Correlations between Psychopathy and Substance Use Abstinence**

| **PCLR vs. Substance Use Abstinence (n=229)** | | | | | |
| --- | --- | --- | --- | --- | --- |
|  | **age** | **IQ** | **PCLR Total** | **PCLR Factor 1** | **PCLR Factor 2** |
| **Alcohol - Abstinence** | .315^**^ | .130 | -.037 | -.119 | .036 |
| **Heroin - Abstinence** | .079 | .133^*^ | .058 | .033 | .070 |
| **Cocaine - Abstinence** | .312^**^ | .078 | .056 | -.085 | .136^*^ |
| **Cannabis - Abstinence** | .306^**^ | .034 | .017 | -.051 | .061 |
| **Meth - Abstinence** | .071 | .121 | .026 | -.037 | .082 |
| **Other Amphetamines - Abstinence** | .075 | .151^*^ | .018 | -.021 | .073 |
| **Nicotine - Abstinence** | .343^**^ | .153^*^ | -.025 | -.126 | .087 |
| **Other Opiates - Abstinence** | .052 | .073 | -.043 | -.006 | -.032 |
| *. Correlation is significant at the 0.05 level (2-tailed). | | | | | |
| **. Correlation is significant at the 0.01 level (2-tailed). | | | | | |
