# Supplementary material for: Abnormal frontostriatal activity in recently abstinent cocaine users during implicit moral processing
Source: Front Hum Neurosci. 2015 Oct 16;9:565. doi: 10.3389/fnhum.2015.00565 (PMC4608360; doi:10.3389/fnhum.2015.00565)
Supplement: Supplementary file 4 [file Table4.DOCX]

**Behavioral Results**

Overall, incarcerated participants responded more slowly when viewing moral pictures (M =1017.47, SD =341.75) than non-moral pictures (M =991.73, SD =322.51), *t*(292) =3.019, *p*=.003. Incarcerated participants also responded more slowly to affectively valenced pictures (moral and non-moral) than neutral pictures (M =910.33, SD =283.13), t(292) =9.230, *p* < .0005.

Non-incarcerated control participants followed this trend for affective versus neutral stimuli, but not for moral versus non-moral stimuli. As a group, these participants responded slightly faster to moral pictures (M=1067.17, SD = 306.16) than non-moral pictures (M = 1093.48, SD= 383.25); a trend which was significantly different from that shown by incarcerated participants, t(327) = 2.002, p = .046. Like the incarcerated population, non-incarcerated participants responded significantly more slowly to affective stimuli than neutral stimuli (M=957.69, SD = 271.59), *t*(35) = 4.130*, p* < .0005.

The CU subset of the incarcerated population also showed little to no difference in response time to moral pictures compared to non-moral pictures (M= -5.27, SD=167.62), which deviated significantly from the NCU group, *t*(291) = 2.283, *p*=.023. Additionally, this difference held when CUs were compared to the mNCU group, t(164) = 2.145, p =.033.

The difference in reaction times to moral pictures compared to non-moral pictures was not significantly correlated with the severity of cocaine use or PCL-R Factor scores.

**Supplementary Table S4a:** Breakdown of Reaction Time Data

**Supplementary Table S4b:** Group Comparisons of Reaction Time Data
